# Genomic analysis of an outbreak of toxin gene bearing *Corynebacterium diphtheriae* in Northern Queensland, Australia reveals high level of genetic similarity

**DOI:** 10.1017/S0950268823000699

**Published:** 2023-05-22

**Authors:** Rikki M. A. Graham, Irani U. Rathnayake, Sumeet Sandhu, Murari Bhandari, Caroline Taunton, Valmay Fisher, Allison Hempenstall, Tonia Marquardt, Amy V. Jennison

**Affiliations:** 1 Public Health Microbiology and Queensland Public Health and Infectious Diseases Reference Genomics (Q-PHIRE Genomics), Forensic and Scientific Services, Queensland Health, Brisbane, QLD, Australia; 2 Public Health Unit, Torres and Cape Hospital and Health Service, Cairns, QLD, Australia; 3 Tropical Public Health Services (TPHS), Cairns, QLD, Australia

**Keywords:** Corynebacterium, molecular biology, molecular epidemiology, outbreaks, public health microbiology

## Abstract

Toxigenic diphtheria is rare in Australia with generally fewer than 10 cases reported annually; however, since 2020, there has been an increase in toxin gene-bearing isolates of *Corynebacterium diphtheriae* cases in North Queensland, with an approximately 300% escalation in cases in 2022. Genomic analysis on both toxin gene-bearing and non-toxin gene-bearing *C. diphtheriae* isolated from this region between 2017 and 2022 demonstrated that the surge in cases was largely due to one sequence type (ST), ST381, all of which carried the toxin gene. ST381 isolates collected between 2020 and 2022 were highly genetically related to each other, and less closely related to ST381 isolates collected prior to 2020. The most common ST in non-toxin gene-bearing isolates from North Queensland was ST39, an ST that has also been increasing in numbers since 2018. Phylogenetic analysis demonstrated that ST381 isolates were not closely related to any of the non-toxin gene-bearing isolates collected from this region, suggesting that the increase in toxigenic *C. diphtheriae* is likely due to the expansion of a toxin gene-bearing clone that has moved into the region rather than an already endemic non-toxigenic strain acquiring the toxin gene.

## Introduction

Toxigenic *Corynebacterium diphtheriae* is the main aetiological agent of diphtheria, which manifests itself as either respiratory or cutaneous disease. Respiratory diphtheria is life-threatening; it is characterised by fever, neck swelling, and sore throat, which can lead to breathing and swallowing difficulties. Cutaneous diphtheria results in non-healing ulcer-like skin lesions and can act as a source of respiratory diphtheria in vulnerable people [1]. Until the establishment of widespread vaccination, diphtheria posed a great threat to public health in Australia [2–5]. The *C. diphtheriae* vaccination utilises the diphtheria toxoid and helps in protection against toxin-associated clinical disease. In Australia, vaccines containing the diphtheria toxin are included in the National Immunisation Program, where doses are funded for children at 2, 4, 6, and 18 months, and again at 4 years of age. Further doses are provided in adolescence as part of school immunisation programmes, as well as for pregnant women, but, although recommended, these are not funded for adults. Vaccination does not prevent the carriage of *C. diphtheriae* but is estimated to reduce onward transmission by 60% and may reduce the risk of severe disease [1].

In 2022, outbreaks of both cutaneous and respiratory diphtheria cases caused by *C. diphtheriae* were reported in some European countries [6, 7]. Genomic analysis illustrated several multi-locus sequence type (MLST) profiles amongst isolates in these outbreaks, suggesting that they are not associated with a single clonal event but rather linked to risk factors including migrant movement and low vaccination rates in particular populations. Similarly, an increase in diphtheria cases caused by multiple genetic lineages of *C. diphtheriae* since 2017 in Yemen has also been reported, where vaccination efforts were disrupted by civil war [8].

As a result of ongoing vaccination efforts in Australia, diphtheria is rare, with generally fewer than 10 cases notified each year. Typically, cases are sporadic and associated with acquisition outside of Australia. There have been fewer than 10 cases of respiratory diphtheria reported in Australia since 2001, with two fatal cases occurring in 2011 and 2018 in the state of Queensland [4, 9, 10]. The detection of the toxin gene in *C. diphtheriae* isolates is notifiable under Queensland public health guidelines [11]; however, non-toxin gene-bearing *C. diphtheriae* is regularly isolated from patients, primarily from wound swabs but also from throat swabs where it is a commensal organism [5, 12]. Whilst occasional detections of toxin gene-bearing *C. diphtheriae* were reported previously, since 2020, North Queensland (NQ) (defined as the region of Queensland north of the tropic of Capricorn) has seen an increase in locally acquired toxin gene-bearing diphtheria cases, with a significant surge during 2022 [13]. Between January and August 2022, 21 patients with a residential address in NQ had toxin gene-positive *C. diphtheriae* isolated compared to 13 patients in the preceding 5 years (2017–2021). A further three interstate cases in 2022 were identified as having epidemiological links to the region.

Here we report on sequence typing of toxin gene-positive *C. diphtheriae* isolates collected in Queensland between January 2017 and August 2022 and investigate the genetic diversity between them and non-toxin gene-bearing isolates recovered in the NQ region during this period.

## Materials and methods

### Isolate collection and DNA extraction

The clinical isolates included in this study are listed in Supplementary Table S1. These comprised a selection of 131 *C. diphtheriae* isolates received by the Queensland Health Public Health Microbiology Reference Laboratory between January 2017 and August 2022, including 51 toxin gene-bearing isolates from across Queensland and 80 non-toxin gene-bearing *C. diphtheriae* from the NQ region. A single toxin gene-bearing ST381 isolate from 2013 was also included in the analysis, bringing the study sample to 132 isolates in total. DNA was extracted from isolates grown overnight at 37°C on horse blood agar (Edwards Group Holdings, Murrarie, Australia), using the QiaSymphony DSP DNA Mini kit (Qiagen, Hilden, Germany), according to the manufacturer’s protocol.

### DNA library and whole genome sequencing

Whole genome sequencing was performed on the isolates listed in Supplementary Table S1 using the procedure described in our previous studies [14, 15]. Briefly, DNA was prepared for sequencing using the Nextera XT kit (Illumina, San Diego, USA) and sequenced on the NextSeq500 using the NextSeq 500 Mid Output v2 kit (300 cycles) (Illumina), according to the manufacturer’s instructions. Sequence reads for the *C. diphtheriae* isolates were trimmed with Trimmomatic v0.36 [16] and quality checked by FastQC v0.11.5 and MultiQC v1.1 [17]. Sequences were *de novo* assembled into contigs using the SPAdes assembler v3.12.0 [18]. Sequence files have been submitted to the European Nucleotide Archive under project accession number PRJEB58646.

### Data analysis

Phylogenetic analysis was performed using single nucleotide polymorphism (SNP)-based typing. Core SNPs were determined for the ST381 reference strain CD38 (SRR6816591, pubMLST id914) with Snippy v4.3.6, using the default settings (available at https://github.com/tseemann/snippy). Core SNPs from each sample were aligned using Snippy-core. A Maximum Likelihood tree was generated from the SNP alignment, using Fast Tree v2.1.10. Interactive tree of life (iTOL) v5.6 was used for the visualization of the phylogenetic tree [19]. MLST was performed in RidomSeqSphere+8.4.1 (Ridom, Munster, Germany) according to the scheme described in [20], available at https://bigsdb.pasteur.fr/diphtheria/. Abricate (https://github.com/tseemann/abricate) was used to search for the presence of acquired antimicrobial resistance genes using the resfinder database [21]. Penicillin-binding protein (*pbp*) genes were investigated using the allele scheme available at https://bigsdb.pasteur.fr/diphtheria/, and by manual interrogation of the *pbp2B* gene for mutations described in [22].

## Results

Of the 51 toxin gene-positive isolates from all Queensland cases between January 2017 and August 2022, 42 (82.3%) were recovered from wound swabs and 9 (17.6%) from throat swabs. Ten (19.6%) of the toxin gene-positive strains were thought to be acquired from outside of Australia based on provided travel history, while the remainder were from cases that had not reported any travel outside of Australia. A single isolate with ST379 had a non-functional toxin gene as previously described [12].

In early 2022, an increase in toxin gene-bearing *C. diphtheriae* cases associated with NQ was observed, with 24 cases identified between January and August 2022 compared to 6 cases in the region in each of 2020 and 2021. The majority of isolates in 2022 were from wound swabs (n = 18), with six from throat swabs. Of these six patients, two presented with classic respiratory diphtheria symptoms, including toxin-mediated complications such as formation of pseudo-membrane, myocarditis, or bulbar palsy, and were treated with antitoxin, while four presented with mild respiratory illness. All 24 cases had epidemiological links to NQ during their exposure period; no international travel nor links to overseas travellers were identified on investigation and all cases were therefore considered locally acquired. Fourteen of the 24 cases were detected following public health interventions; 2 were close contacts and 1 was a casual contact (identified through contact tracing), with another 11 cutaneous cases detected through enhanced clinical surveillance in an area where cases had occurred.

### MLST analysis

MLST analysis of the 132 *C. diphtheriae* isolates (80 non-toxin gene-bearing isolates from the NQ region and 52 toxin gene-bearing isolates from across Queensland) identified 27 different sequence types (STs). Toxin gene-positive strains isolated prior to 2020 exhibited a range of different STs; however, from January 2020 to August 2022, 29/34 (88.3%) of the toxin gene-positive isolates were ST381. This ST had been relatively rare in Queensland before this time, with only three representatives being isolated prior to 2020, and all were associated with travel to islands in the southwestern Pacific. Three ST381 isolates were identified in 2020 and three in 2021 (Supplementary Table S1). Numbers of ST381 *C. diphtheriae* increased markedly in 2022 with 23 isolated between January and August 2022. All of these ST381 isolates were recovered from patients who either were residents of NQ or had epidemiological links to this region.

### Phylogenetic analysis

**Figure 1.**
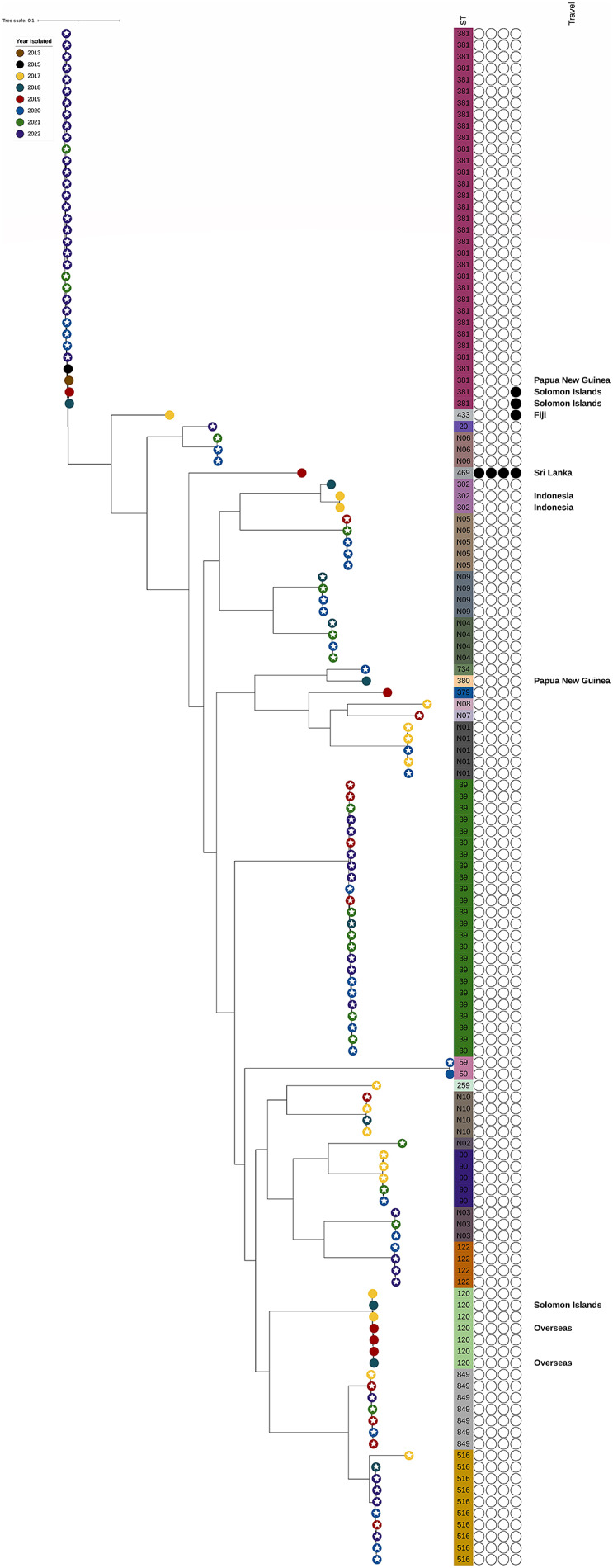
Maximum likelihood tree built using SNP differences between *Corynebacterium diphtheriae* isolates. Colours of leaves represent the year isolated and white stars on leaves indicate that the isolate was from the NQ region. ST and AMR genes are indicated to the right of the tree. Branch length represents genetic distance as indicated by the scale bar.

**Figure 2.**
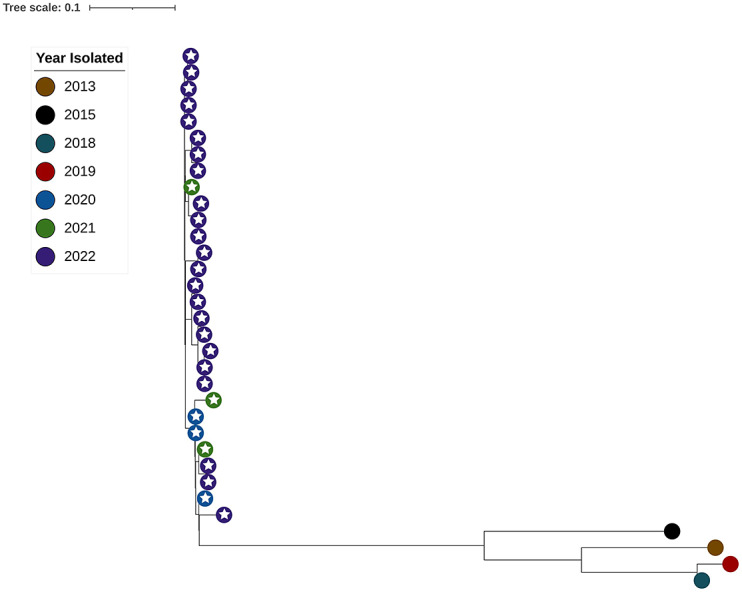
Maximum likelihood tree built using SNP differences between ST381 *Corynebacterium diphtheriae* isolates. Colours of leaves represent the year isolated and white stars on leaves indicate that the isolate was from the NQ region. Branch length represents genetic distance as indicated by the scale bar.

When all sequenced isolates were analysed by SNP analysis, clustering based on STs was observed, with the ST381 clone forming a group distinct from the other STs included in the analysis (Figure 1). The analysis showed that the ST381 isolates from 2020 to 2022 were more closely related to each other than to ST381 cases from before 2020, with more than 120 SNPs present between the pre-2020 isolates and the 2020–2022 isolates (Figure 2). Among the 2020–2022 ST381 isolates, there were fewer than 20 SNPs present between isolates, with no temporal differences in clustering (isolates from different years showed the same number of SNPs between them as isolates from the same year). Further analysis of SNP differences found that none of the other ST groups were as tightly clustered as the ST381 group.

The *C. diphtheriae* MLST Institut Pasteur database contains information from two isolates of ST381, one being a Queensland isolate from 2013 included in this study and the other being grown from a cutaneous case in the state of New South Wales (NSW), Australia, from 2015 (id914 used as the reference in the phylogenetic analysis). BURST analysis of the ST381 profile in the *C. diphtheriae* MLST Institut Pasteur database revealed that the closest sequence type to ST381 is the single locus variant (SLV) ST461. Only one ST461 is in the MLST database – a cutaneous case from 2016 also from NSW.

**Figure 3.**
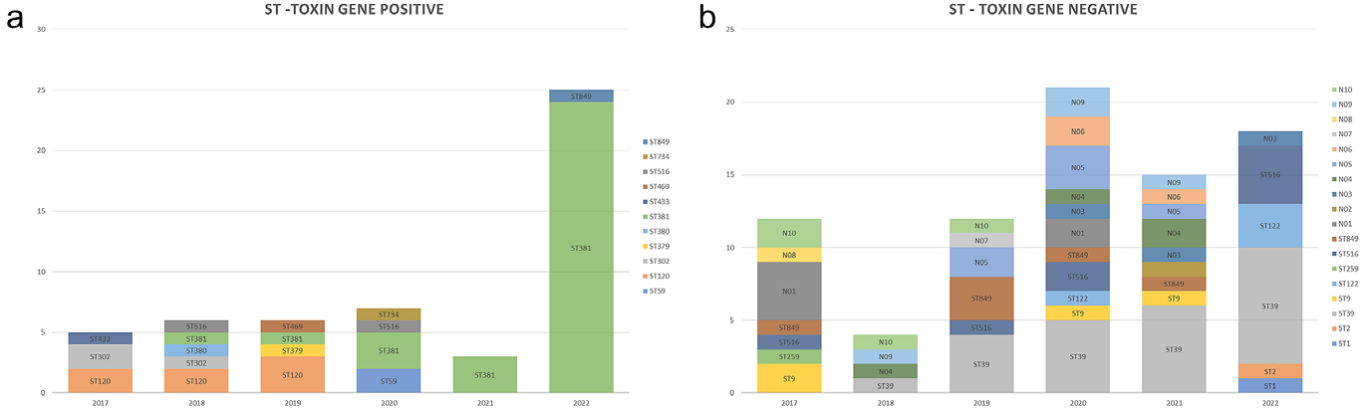
Numbers of isolates with different STs by year in (a) toxin gene-positive and (b) toxin gene-negative isolates. STs are shown on the columns as well as in the legend for clarity.

Other than ST381, the most common ST of isolates from the NQ region was ST39. These strains were toxin gene negative but have been increasingly isolated since 2018, with numbers rising from one in 2018 to eight in 2022 (Figure 3).

**Figure 4.**
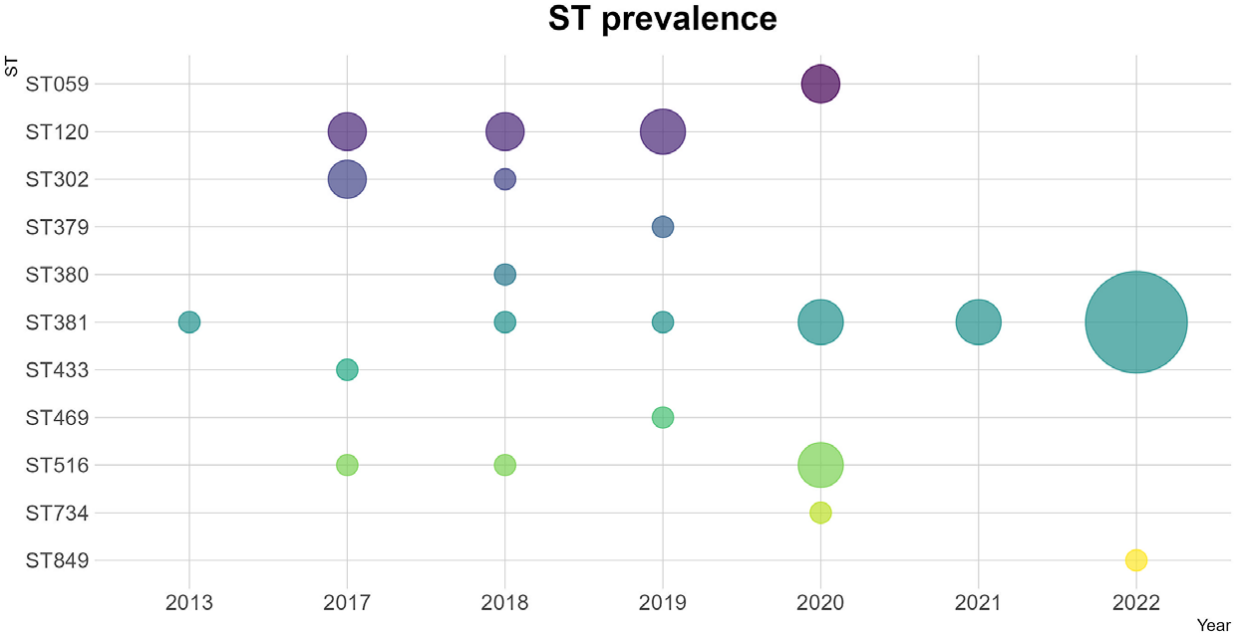
Prevalence of STs per year for toxin gene-bearing *Corynebacterium diphtheriae* isolates. Circle size represents number of isolates and colours represent ST.

The prevalence of different STs among toxin gene-positive strains detected in Queensland appears to have changed between 2017 and 2022, with some STs present prior to 2022 becoming less frequent, and a decrease in the diversity of STs seen in 2021 and 2022 when compared to previous years (Simpson’s diversity index of 0.07 for 2021–2022 compared to 0.88 for 2017–2020). ST381 was the only lineage among toxin gene-bearing strains between January 2021 and August 2022, accounting for all but one of 27 (96.3%) such strains from the NQ region (Figure 4).

### AMR genes

Screening of the sequences for acquired AMR genes identified four isolates with *sul1*, and one isolate with *aph(3′), aph(6)-Id, cmx1* and *sul1* (Figure 1).

Screening of sequences for *pbp* revealed the presence of *pbp1A*, *pbp1B*, *pbp2A*, *pbp2B*, *pbp2C*, and *pbp4* with alleles as shown in Supplementary Table S1. The *pbp2B* mutation V535I and the *pbp2m* gene were not found in any of the isolates.

## Discussion

Phylogenetic analysis of *C. diphtheriae* from the NQ region between January 2017 and August 2022 has demonstrated that the genetic population of *C. diphtheriae* in this region has changed over time, with STs such as N01 and N05 decreasing and ST39 increasing in the non-toxin gene-bearing *C. diphtheriae* population, and ST381 emerging as dominant in the toxin gene-bearing population. Isolates of both ST39 and ST381 increased markedly in 2022. At a genomic level, ST39 is not closely related to ST381; so despite their increase in numbers at the same time in the same region, it is unlikely that they are genetically linked, and these increases possibly represent changes in the bacterial population over time. This change is potentially in response to a decrease in the introduction of international strains due to public health interventions in Australia during the COVID-19 pandemic, including border restrictions on international arrivals, which may have allowed local strains to expand. However, as we have only sequenced the non-toxigenic strains from NQ, we cannot comment on whether similar increases in ST39 have occurred in other regions of Queensland, or more widely in Australia.

The STs seen in the toxin gene-positive isolates included in this study differ from the globally dominant STs reported, and in contrast to the increases in *C. diphtheriae* incidence reported in other countries, which have been polyclonal in nature with a range of STs being reported [2, 6–8, 23], the increased incidence of toxin gene-positive *C. diphtheriae* in NQ appears primarily linked to the clonal expansion of ST381 alone. The reason for this is not known; however, the tropical environment of NQ and social determinants, including household overcrowding in the areas where most diphtheria cases have been reported, may be contributing to transmission. Public health interventions in the NQ region in response to the ST381 clonal expansion are continuing.

All three ST381 cases identified in Queensland prior to 2020 were associated with travel to the southwest Pacific islands. Travel history is not documented for the single ST381 case listed in the *C. diphtheriae* MLST Institut Pasteur database, nor for the isolate of the nearest sequence type, ST461, but both originated from NSW, Australia, in 2015–2016. Our phylogenetic analysis suggests that during or prior to 2020, a particular clone of ST381, which does not appear closely genetically linked to any of the previous ST381 detections, has rapidly expanded in the NQ region, resulting in a cluster of locally acquired toxin gene-bearing cutaneous and respiratory diphtheria cases. There is no evidence of sustained circulation of toxigenic *C. diphtheriae* in Australia since the early 1990s [24].

A difference in ST prevalence was seen between the NQ non-toxin gene-bearing strains and those reported in another Australian study, where ST32 was the most commonly reported non-toxin gene-bearing ST in the state of NSW, which is located to the south of Queensland [5]. It may be that in the more urban southern region of Queensland, the *C. diphtheriae* population is similar to that seen in the southern Australian states and this would make an interesting future study.

Toxin gene-positive and non-toxin gene-bearing strains tended to fall into different STs (Figure 1); however, ST849 and ST516 contained both toxigenic and non-toxigenic strains. This may represent loss or acquisition of the diphtheria toxin phage by these strains, but overall, the genomic results indicate that for the most part, toxigenic and non-toxigenic strains are genomically distinct. The presence of the toxin gene in all ST381 isolates included in this study suggests that clonal expansion of this strain across NQ between 2020 and 2022 did not arise from the local acquisition of the toxin phage by a non-toxigenic strain. Although the production of diphtheria toxin by the toxin gene-positive isolates was not confirmed phenotypically as the Elek test is no longer carried out by any Australian laboratory, a correlation between the presence of the toxin gene and the production of the toxin has been described [25].

The presence of antimicrobial resistance markers was not common in the strains analysed, with only four containing genes associated with resistance, all of which were toxin gene positive and from cases who had reported travel. One of these strains (M1911202 and ST469 (Supplementary Table S1)) contained multiple resistance genes, associated with resistance to macrolides, chloramphenicol, and sulfonamides; however, unfortunately, information on the potential phenotypic resistance was not available. This level of antimicrobial resistance is lower than that seen in other countries [22, 26]. In 2011, there was a fatal case of respiratory *C. diphtheriae* in Queensland caused by a penicillin-resistant strain carrying the *pbp2m* gene [9, 10, 22]. Although several *pbp* genes were found in the isolates analysed in this study, none had the *pbp2m* gene present or the V535I mutation in the *pbp2B* gene that is associated with resistance to penicillin [22].

This study reports on a spatially and temporally related increase in toxin gene-positive *C. diphtheriae* cases in NQ, first noted in 2021 by both relevant public health authorities and the Queensland microbiology reference laboratory. A source of possible bias of this study is that some of the recent increase in toxin gene-positive *C. diphtheriae* isolates may have been driven by the increased health surveillance in this region leading to an increase in testing and reporting of diphtheria, and increased isolation of diphtheroids that may have previously been dismissed as skin flora.

## Conclusions

In conclusion, this genomic analysis of *C. diphtheriae* has demonstrated that an increase in cases of toxin gene-bearing *C. diphtheriae* in NQ is likely due to an increase in one ST, ST381. This group clusters closely together with higher genetic relatedness amongst all 2020–2022 ST381 isolates than to ST381 isolates from previous years and is not closely related to non-ST381 isolates or any of the non-toxin gene-bearing strains. Thus, this likely represents the clonal expansion of a toxin gene-bearing strain that has moved into this region rather than the result of an already endemic non-toxigenic strain acquiring the toxin gene.

## Data Availability

Sequence files have been submitted to the European Nucleotide Archive under project accession number PRJEB58646.

## Supplementary material

For supplementary material accompanying this paper visit http://doi.org/10.1017/S0950268823000699. click here to view supplementary material

## References

[1] Truelove SA, Keegan LT, Moss WJ, Chaisson LH, Macher E, Azman AS and Lessler J (2020) Clinical and epidemiological aspects of diphtheria: A systematic review and pooled analysis. Clinical Infectious Diseases 71(1), 89–97. 31425581 10.1093/cid/ciz808 PMC7312233

[2] Meinel DM, Kuehl R, Zbinden R, Boskova V, Garzoni C, Fadini D, Dolina M, Blümel B, Weibel T, Tschudin-Sutter S, Widmer AF, Bielicki JA, Dierig A, Heininger U, Konrad R, Berger A, Hinic V, Goldenberger D, Blaich A, Stadler T, Battegay M, Sing A and Egli A (2016) Outbreak investigation for toxigenic *Corynebacterium diphtheriae* wound infections in refugees from northeast Africa and Syria in Switzerland and Germany by whole genome sequencing. Clinical Microbiology and Infection 22(12), 1003.e1001–1003.e1008. 10.1016/j.cmi.2016.08.010 27585943

[3] Adler NR, Mahony A and Friedman ND (2013) Diphtheria: Forgotten, but not gone. Internal Medicine Journal 43(2), 206–210. 23402486 10.1111/imj.12049

[4] Winkler NE, Dey A, Quinn HE, Pourmarzi D, Lambert S, McIntyre P and Beard F (2022) Australian vaccine preventable disease epidemiological review series: Diphtheria 1999–2019. Communicable Diseases Intelligence (2018) 46. 10.33321/cdi.2022.46.42 35860872

[5] Timms VJ, Nguyen T, Crighton T, Yuen M and Sintchenko V (2018) Genome-wide comparison of *Corynebacterium diphtheriae* isolates from Australia identifies differences in the pan-genomes between respiratory and cutaneous strains. BMC Genomics 19(1), 869. 30509172 10.1186/s12864-018-5147-2 PMC6278121

[6] Kofler J, Ramette A, Iseli P, Stauber L, Fichtner J, Droz S, Zihler Berner A, Meier AB, Begert M, Negri S, Jachmann A, Keller PM, Staehelin C and Grützmacher B (2022) Ongoing toxin-positive diphtheria outbreaks in a federal asylum centre in Switzerland, analysis July to September 2022. Eurosurveillance 27(44), 2200811. 36330823 10.2807/1560-7917.ES.2022.27.44.2200811 PMC9635023

[7] Badenschier F, Berger A, Dangel A, Sprenger A, Hobmaier B, Sievers C, Prins H, Dörre A, Wagner-Wiening C, Külper-Schiek W, Wichmann O and Sing A (2022) Outbreak of imported diphtheria with *Corynebacterium diphtheriae* among migrants arriving in Germany, 2022. Eurosurveillance 27(46), 2200849. 36398576 10.2807/1560-7917.ES.2022.27.46.2200849 PMC9673234

[8] Badell E, Alharazi A, Criscuolo A, Almoayed KAA, Lefrancq N, Bouchez V, Guglielmini J, Hennart M, Carmi-Leroy A, Zidane N, Pascal-Perrigault M, Lebreton M, Martini H, Salje H, Toubiana J, Dureab F, Dhabaan G, Brisse S and NCPHL Diphtheria Outbreak Working Group (2021) Ongoing diphtheria outbreak in Yemen: A cross-sectional and genomic epidemiology study. The Lancet Microbe 2(8), e386–e396. 35544196 10.1016/S2666-5247(21)00094-X

[9] Forde BM, Henderson A, Playford EG, Looke D, Henderson BC, Watson C, Steen JA, Sidjabat HE, Laurie G, Muttaiyah S, Nimmo GR, Lampe G, Smith H, Jennison AV, McCall B, Carroll H, Cooper MA, Paterson D and Beatson SA (2020) Fatal respiratory diphtheria caused by ß-lactam-resistant *Corynebacterium diphtheriae*. Clinical Infectious Diseases 73(11), e4531–e4538. 10.1093/cid/ciaa1147 32772111

[10] Grigg S, Hogan D, Hosein FS, Johns D, Jennison A and Subedi S (2020) A case of toxigenic, pharyngeal diphtheria in Australia. Medical Journal of Australia 213(2), 64–65.e61. 32227479 10.5694/mja2.50566

[11] QLD Department of Health (2023) *Diphtheria – Queensland Health Guidelines for Public Health Units.* Available at https://www.health.qld.gov.au/cdcg/index/diphtheria.

[12] Doyle CJ, Mazins A, Graham RMA, Fang NX, Smith HV and Jennison AV (2017) Sequence analysis of toxin gene-bearing *Corynebacterium diphtheriae* strains, Australia. Emerging Infectious Diseases 23(1), 105–107. 27983494 10.3201/eid2301.160584 PMC5176206

[13] Hempenstall A, Short J, Marquardt T, Fisher V and Johnson J (2023) Clinician alert: Toxigenic diphtheria cases across north Queensland are on the rise. Medical Journal of Australia 218(5), 238. 10.5694/mja2.51858 36806213

[14] Graham RMA, Hiley L, Rathnayake IU and Jennison AV (2018) Comparative genomics identifies distinct lineages of S. Enteritidis from Queensland, Australia. PLoS One 13(1), e0191042. 29338017 10.1371/journal.pone.0191042 PMC5770046

[15] Pintara A, Jennison A, Rathnayake IU, Mellor G and Huygens F (2020) Core and accessory genome comparison of Australian and international strains of O157 Shiga toxin-producing *Escherichia coli*. Frontiers in Microbiology 11, 566415. 33013798 10.3389/fmicb.2020.566415 PMC7498637

[16] Bolger AM, Lohse M and Usadel B (2014) Trimmomatic: A flexible trimmer for Illumina sequence data. Bioinformatics 30(15), 2114–2120. 24695404 10.1093/bioinformatics/btu170 PMC4103590

[17] Wingett SW and Andrews S (2018) Fastq screen: A tool for multi-genome mapping and quality control. F1000Research 7, 1338. 30254741 10.12688/f1000research.15931.1 PMC6124377

[18] Bankevich A, Nurk S, Antipov D, Gurevich AA, Dvorkin M, Kulikov AS, Lesin VM, Nikolenko SI, Pham S, Prjibelski AD, Pyshkin AV, Sirotkin AV, Vyahhi N, Tesler G, Alekseyev MA and Pevzner PA (2012) Spades: A new genome assembly algorithm and its applications to single-cell sequencing. Journal of Computational Biology 19(5), 455–477. 22506599 10.1089/cmb.2012.0021 PMC3342519

[19] Letunic I and Bork P (2019) Interactive tree of life (iTOL) v4: Recent updates and new developments. Nucleic Acids Research 47(W1), W256–W259. 30931475 10.1093/nar/gkz239 PMC6602468

[20] Bolt F, Cassiday P, Tondella ML, DeZoysa A, Efstratiou A, Sing A, Zasada A, Bernard K, Guiso N, Badell E, Rosso ML, Baldwin A and Dowson C (2010) Multilocus sequence typing identifies evidence for recombination and two distinct lineages of *Corynebacterium diphtheriae*. Journal of Clinical Microbiology 48(11), 4177–4185. 20844217 10.1128/JCM.00274-10 PMC3020856

[21] Chen L, Zheng D, Liu B, Yang J and Jin Q (2016) VFDB 2016: Hierarchical and refined dataset for big data analysis – 10 years on. Nucleic Acids Research 44(D1), D694–697. 26578559 10.1093/nar/gkv1239 PMC4702877

[22] Hennart M, Panunzi LG, Rodrigues C, Gaday Q, Baines SL, Barros-Pinkelnig M, Carmi-Leroy A, Dazas M, Wehenkel AM, Didelot X, Toubiana J, Badell E and Brisse S (2020) Population genomics and antimicrobial resistance in *Corynebacterium diphtheriae*. Genome Medicine 12(1), 107. 33246485 10.1186/s13073-020-00805-7 PMC7694903

[23] Guglielmini J, Hennart M, Badell E, Toubiana J, Criscuolo A and Brisse S (2021) Genomic epidemiology and strain taxonomy of *Corynebacterium diphtheriae*. Journal of Clinical Microbiology 59(12), e0158121. 34524891 10.1128/JCM.01581-21 PMC8601238

[24] Patel M, Morey F, Butcher A, Moore C, Brennan R and Mollison L (1994) The frequent isolation of toxigenic and non-toxigenic *Corynebacterium diphtheriae* at Alice Springs Hospital. Communicable Diseases Intelligence 18(13), 310–311.

[25] Hauser D, Popoff MR, Kiredjian M, Boquet P and Bimet F (1993) Polymerase chain reaction assay for diagnosis of potentially toxinogenic *Corynebacterium diphtheriae* strains: Correlation with ADP-ribosylation activity assay. Journal of Clinical Microbiology 31(10), 2720–2723. 8253972 10.1128/jcm.31.10.2720-2723.1993 PMC265991

[26] Husada D, Soegianto SDP, Kurniawati IS, Hendrata AP, Irawan E, Kartina L, Puspitasari D, Basuki PS and Ismoedijanto (2019) First-line antibiotic susceptibility pattern of toxigenic *Corynebacterium diphtheriae* in Indonesia. BMC Infectious Diseases 19(1), 1049. 31829153 10.1186/s12879-019-4675-y PMC6907133

